# Targeting Viral ORF3a Protein: A New Approach to Mitigate COVID-19 Induced Immune Cell Apoptosis and Associated Respiratory Complications

**DOI:** 10.34172/apb.2023.069

**Published:** 2023-01-23

**Authors:** Minu Treeza M, Sanu Augustine, Aparna Ann Mathew, S.K. Kanthlal, Rajitha Panonummal

**Affiliations:** Amrita School of Pharmacy, Amrita Institute of Medical Sciences & Research Centre, Amrita Vishwa Vidyapeetham, Kochi-682041, India.

**Keywords:** SARS-CoV-2, COVID-19, Mutation, Viral variants, Apoptosis, ORF3a

## Abstract

Infection with SARS-CoV-2 is a growing concern to the global well-being of the public at present. Different amino acid mutations alter the biological and epidemiological characteristics, as well as immune resistance of SARS-CoV-2. The virus-induced pulmonary impairment and inflammatory cytokine storm are directly related to its clinical manifestations. But, the fundamental mechanisms of inflammatory responses are found to be the reason for the death of immune cells which render the host immune system failure. Apoptosis of immune cells is one of the most common forms of programmed cell death induced by the virus for its survival and virulence property. ORF3a, a SARS-CoV-2 accessory viral protein, induces apoptosis in host cells and suppress the defense mechanism. This suggests, inhibiting SARS-CoV-2 ORF3a protein is a good therapeutic strategy for the treatment in COVID-19 infection by promoting the host immune defense mechanism.

## Introduction

 Coronavirus is an enclosed, positive-stranded RNA virus with a large genome, ranging from 27 to 32 kb, and can infect mammals, birds, humans, causing both acute and chronic illnesses.^1^ Members of this family have been identified as the causal agents of infectious bronchitis in chickens since the 1930s.^2^ Coronavirus is a member of *Coronaviridae* family, *Cornidovirineae* suborder, and of the order *Nidovirales.*^3^ Coronaviruses are divided into four genera, namely Alfa, Beta, Gamma and Delta.^1^ All five subgenera of the genus *Betacoronavirus*, i.e. *Embevovirus, Merbecovirus, Nobecovirus, Hibecovirus* and *Sarbecovirus* were found in a bat-derived coronavirus., according to phylogenetic analysis.^4^ A new coronavirus, SARS-CoV-2, caused an outbreak in Wuhan, China, in December 2019.^5^ In the subgenus *Sarbecovirus*, bat-derived coronaviruses bat-SL-CoVZXC21 and bat-SL-CoVZC45, with SARS-CoV-2 being the most likely to be related to these, both of which were discovered in bats.^4^ The *Betacoronavirus* includes MERS-CoV, SARS-CoV and now SARS-CoV-2.^1,6^

 MERS-CoV is a type of coronavirus capable of infecting humans that was firstly detected in Saudi Arabia, September 2012.^7^ Viral membrane fusion and entry into a host cell occurs as a result of the binding of envelope S glycoprotein to the dipeptidyl peptidase 4 (DPP4) cellular receptor and releasing the viral RNA genome to the cytoplasm via endosome formation or direct membrane entry.^8^ Unlike MERS-CoV, the membrane fusion and entry of SARS-CoV in to host cell occurs by attachment of S glycoprotein to human angiotensin converting enzyme 2 (hACE2), which is expressed highly in the epithelial cells of lungs, small intestine, and is also found in skeletal muscles and the brain.^6,8,9^ The antigen and viral kinetic studies of SARS-CoV carried out in transgenic mice showed that the viral infection originated from the epithelium of respiratory tract, quickly reached the alveoli and was spread to the brain.^9^ Genetic evidence strongly suggests that, SARS-CoV-2 most likely originated in animals.^10^ Genomically, SARS-CoV-2 has 79.5% of sequence similarity with SARS-CoV, 96% with bat coronavirus and 50% with MERS-CoV.^10,11^ The receptor-binding domain (RBD) of SARS-CoV-2 shows similar structure to SARS-CoV in homology modelling, and there are a few differences in critical residues at the amino acid level.^12^ Fever, Pneumonia, mild cough and failure of multiple organ functions, with 2-4% of mortality rate are the main clinical manifestations of SARS-CoV-2 involved viral infections. Anosmia, dysgeusia, and some neurological symptoms have been reported as well.^11^ According to current knowledge, SARS-CoV-2 has a similar transmission mode between humans via droplets, whereas direct or indirect contact with mucus membranes in the mouth, eye, or nose is vulnerable to its transmission.^12^

 Viruses are one of the abundant organisms in the environment with lack of their own metabolic process and therefore rely on a host cell to produce new products.^13,14^ Successful viral replication necessitates not only just effective offspring production and distribution, but also evading host defence mechanisms that limit replication. Some viruses appear to use or manipulate apoptosis as a cell-killing and virus-spreading mechanism.^15^ Many viruses encode proteins that suppress apoptosis, extending the survival of infected cells and increasing the production of progeny virus, or allowing the formation of virus persistence.^16^ So, the infected host cell’s apoptosis has been recognised as a powerful method for limiting viral transmission.^17^

 COVID-19, a global epidemic caused by the SARS-CoV-2 virus, causes severe respiratory illness in those who are infected. Recent studies found that SARS-CoV-2 encoded ORF3a, an accessory protein which can efficiently induce cell apoptosis and play an important role in infection.^14,16^ Throughout this review, we emphasized the present status of researches on the SARS-CoV-2 virus, their variants and suggesting how this information can be apply for future research and innovative therapies in virus induced cell death.

## SARS-CoV-2: genomic characteristics, viral entry and it’s protein-protein interactions

 SARS-CoV-2 possesses a complex genome similar to other *Betacoronavirus*, with two open-reading frames (ORFs): ORF1a as well as ORF1b, coding for nonstructural proteins (NSPs), additional structural proteins, transcriptional factors and viral regulators.^18–20^ ORF1a generates polypeptide 1a (pp1a) with a molecular mass of 440–500 kDa, which will then be cleaved into 11 NSPs. Due to a ribosomal frameshift proximal to ORF1b’s stop codon, ORF1b is translated as part of a larger polypeptide (pp1ab). As a result, a bigger polypeptide of about 800 kDa is generated, which is fragmented into 15 nucleotides by the viral proteases. The viral RNA-dependent RNA polymerase, or NSP12, is another significant NSP that is required for transcription and replication of virus. Positive sense genomic RNA (gRNA) and sub-genomic RNA are transcribed from negative-sense RNA species in the viral genome (sgRNA).^19^

 The structural proteins encoded by sgRNAs are Spike (S), Membrane (M), Envelope (E) nucleocapsid (N) as well as a few auxiliary proteins.^18-20^ The surface-exposed S glycoprotein, which forms homotrimers emerging from the viral surface, aids corona virus entry into host cells. It can be used as a target to develop therapeutics and vaccines. Host proteases degrade S protein into 2 key subunits, S1 and S2, which are prerequisites in the ACE2 receptor binding and fusion of the viral membrane.^21,22^ An amino-terminus, a transmembrane domain, and a carboxy-terminus make up the E protein. It is found in the endoplasmic reticulum and Golgi, where it aids the virus’s assembly, release, and other intracellular activities. According to research, the E protein is thought to be involved in a number of aspects of viral replication. The N protein, which is involved in replication and other cellular responses, is another important structural protein that adheres to the viral genome. M protein aids in structuring the viral envelope and interacts with other structural proteins, such as N protein, which is required for nucleocapsid and S protein assembly for viral integration.^23,24^

 As a functional receptor, SARS-CoV-2 uses ACE2 for viral entry and an enzyme type II serine transmembrane protease 2 (TMPRSS2), for priming the surface-bound S protein.^25,26^ ACE2 is a protein that can be found all over the body including nasal-oral mucosa, stomach, lung, colon, small intestine, skin, lymph nodes, bone marrow, thymus, spleen, liver, kidney, and brain. In addition, ACE2 is found in high concentrations not only in the epithelia of the small intestine and lungs, but also in arterial and venous endothelial cells (ECs) of all organs.^27^ Extra-pulmonary manifestations and multi-organ failures are confirmed in COVID-19 patients, according to these findings. Attachment of the S protein to ACE2 results in viral entry and subsequent membrane fusion with the host cell.^21^ Entered virus will then release its genomic mRNA into the cytoplasm, where it will be translated into viral proteins.

**Figure 1:**
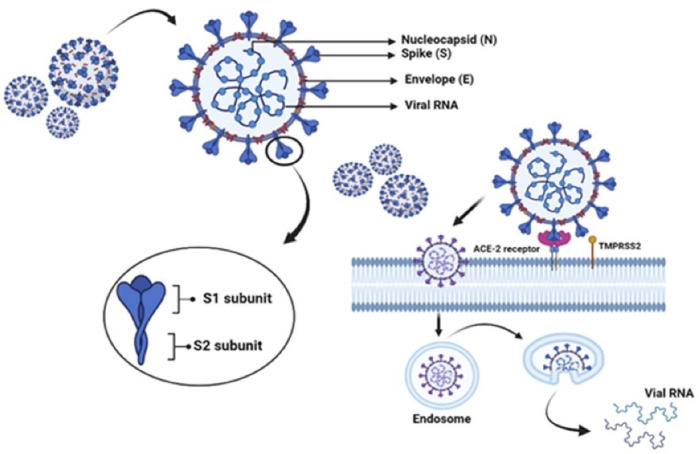
Spike (S), Membrane (M), Envelope (E) and nucleocapsid (N) are the structural proteins of SARS-CoV-2. It enters the host cell by S1-ACE2 interaction in the cell membrane followed by the cleavage of S1 and S2 by TMPRSS2. The entered virus releases its genomic mRNA into the host cell and is transcribed into viral proteins

 S proteins are found on the surface of Coronaviruses as trimers, with each monomer having two domains, S1 and S2. S1 interacts with ACE2 receptor, whereas the membrane fusion is facilitated by S2 domain. The interaction with ACE2 receptors is mediated by RBD located on S1 domain (Figure 1).^28^ It has been demonstrated in an *in vitro* study that SARS-CoV-2 is unable to infect ACE2 knockout Vero E6 and Hela cells, suggesting its importance in viral entry.^28,29^ The cleavage of ACE2 and S-proteins for the entry of virus is mediated by TMPRSS2 enzyme, via membrane fusion, which is a major factor in the infection of COVID-19. In addition, other cell surface components also help Coronaviruses to enter the cells like DPP4 and furin, as in case of MERS-CoV.^28^

 ACE2 is the receptor for S-protein of SARS-CoV, placed on chromosome Xp22.2, encoding the protein that is belonging to the group of angiotensin-converting enzyme. The ACE2 receptor typically remains as a dimer with A and B forms, which is stabilised by the Sodium-dependent neutral amino acid transporter (B^0^ AT1), also called SLC6A19. But it does not support ACE2 dimerization.^30^ ACE2 receptor consists of a peptidase domain (PD) at the N-terminus and a collectrin-like domain at the C-terminus (CLD). 597 amino acids (residues 19–615) constitute PD whereas CLD has 153 amino acids (residues 616–768). CLD aids in the dimerization of ACE2, where the residues, Arg710, Arg652, Ser709, Asp713 and, Glu653 of ACE2-A interact with the residues, Asn638, Asn636, Tyr641, Glu639, Tyr633 and Arg716 of ACE2-B.^31^

 The interface between ACE2 and S-protein within the PD region can be categorized into hydrophobic and H-bonding components. These components aid in the interaction of ACE2 with S-protein. This region enables binding of RBD of the viral S-protein.^26,30^ Within the RBD, residues from Leu335 to Phe515 of SARS-CoV-2 are homologous to the residues from Leu322 to Phe501 of SARS-CoV, except for the insertion of Val483 in SARS-CoV-2. This region has three areas of ACE2 interaction, CR1, CR2 and CR3, derived from a receptor binding motif (RBM) of SARS-CoV-2 having 50% sequence homology to SARS-CoV.^32^ Two S-protein trimers binds to a homodimer of ACE2, each through an ACE2 monomer.^28^ Electrostatic, hydrogen bonding, and hydrophobic interactions of Lys314 and Glu54 of the ACE2 receptor with the S1 domain are reported in studies using molecular docking models.^33^ Three-dimensional electron microscopy reconstruction of the RDB-ACE2-B^0^ AT1 complex assisted to identify the specific molecular interactions. H-bonds are formed between the amino acids Thr500, Asn501and Gln498 of RDB and the amino acids Tyr4, Arg357 and Structural basis for the recognition of SARS-CoV-2 by full-length human ACE2 contacts between the Lys417, Tyr453, Gln474, and Phe486 residues of RDB and the Gln24, Asp30, and His34 residues of ACE2.^34^

## SARS-CoV-2 and its variants

 All the viruses including corona viruses exhibit continual adaptive changes in the human population.^35,36^ During SARS-CoV-2 replication, mutations may occur spontaneously.^37,38^ However, most mutations have no discernible impact on the virus transmission and pathogenicity, and thus on the disease course.^37,39^ It is possible that new mutations could encourage the spreading of infection which creates a scenario where the available vaccine become ineffective. This occurs mainly due to viral signals that may evade immune defences following previous infections or immunisation.^37,40^ At least 23 ORFs are found in the SARS-CoV-2 virus genome as compared with other corona viruses and its genome comprises ORFs that encode structural proteins and NSPs. Various mutations in the S gene, which is involved in viral entry into cells, have the greatest impact. SARS-CoV-2 variants with major S protein mutations, and their role in progression of infection is summarized in Table 1. S protein has 2 subdomains, S1 and S2, both having a role in viral infection. Both the NTD and CTD of S1 act as RBD and have the capacity to bind with different proteins as well as sugars, and also to hACE2 receptor. Both S1 as well as S2 facilitates the fusion by making conformational changes. So, the mutation in RBD of circulating virus alter the affinity for the receptor and make them more transmissible.^37^ B.1.1.7, B.1.351, P.1, B.1.617.2 and B.1.1.529 are the variants of concern (VOCs) based on the Pango SARS-CoV-2 lineage nomenclature, which implies viral variant with mutations in their RBD altering the binding to hACE2 receptor.^41,42^ According to WHO, the rapidly spreading viral variants are categorized as Alfa, Beta, Gamma, Delta and Omicron.^37,43^

**Table 1:** S Protein mutations and VOC

**Mutations**	**Location**	**Variants**	**Characteristics**
*N501Y*	RBD	Alpha (B.1.1.7)	Binding tightness of N501Y‐RBD to ACE2 raised 10-fold.
		Beta (B.1.351)	Increased the hACE2 binding affinity of RBD.
		Gamma (P.1)	
*L452R*	RBM	Delta (B.1.617.2)	Increased expression of S protein and enhanced the infectivity of the virus.
*L452R ‐ E484Q*	RBM		Enhanced hACE2 binding affinity.
*HV69*⁃*70del*	NTD	Alfa (B.1.1.7)	Accelerates cell-cell fusion and results in the production of multinucleated cells.
*N501Y*			More infectious by rapid S1 and S2.
*N439*			Enhanced S protein binding affinity towards hACE2 and escape from immune response.
*Y453F*			
*T478K*	S protein	Delta (B.1.617.2)	Augment the steric and electrostatic hindrance of spik protein and aids easier RBD binding to ACE2.
*P681H/R*	The furin protease cleavage sites located between S1 and S2 subunits of S protein.	ALFA (B.1.1.7) Delta (B.1.617.2)	S1/S2 cleavage by furin‐like proteases is increased and facilitate the viral attachment to host cells membrane.
*P681H*			
*P681R*			

###  Alfa (B.1.1.7)

 The VOC B.1.1.7 of SARS-CoV-2 is also known as VOC202012/01 and 20I/501Y.VI has emerged in the south east region of England in September 2020 and became the most rapidly circulating strain. After a short period of time, this strain has been detected over 30 countries. Several nonsynonymous substitutions have been identified in B.11.7, including deletions at amino acid position 69 and 70 (60-70) of S protein and replacement at position 501Y. The N501Y replacement results in increased ACE2 binding affinity and its rapid spreading rate. The RT-PCR failed to diagnose this variant, which is related to the 60-70 deletion. This variant has 13 other mutations also.^37,44,45^

###  Beta (B.1.351)

 After the first outbreak wave of the disease, the B.1.351, also called as 501Y.V2 in GH clade which was reported first in South Africa. Apart from D614G, there are 9 mutations of S protein in the B.1.351 genome where two of them are found in NTD (like 242-244 del and R246I) and three in RBD (K417N, E484K, and N501Y), as well as one near the furin cleavage site (A701V). Several mutations including those in the antigenic supersite of NTD or in the ACE2 binding site are occur in this lineage. These mutations may impair the effectiveness of monoclonal antibody therapy or vaccination.^37,46^ Among the 3 mutations in RBD, E484K and N501Y are within the RBM, which forms the interface within hACE2 receptor. N501 is a component of the binding loop found in the contact area of hACE2 that forms a H-bond with Y41. Additionally, it stabilizes the K353 residue, a virus-binding hotspot for hACE2. E484 existing in the RBM interacts with the K31 interaction hotspot residue of hACE2. K417 is a newly discovered interacting residue with hACE2 which interacts with D30 of hACE2 across the central contact region. Here E484K, and N501Y showing enhanced binding affinity to hACE2 whereas minimal affinity in the case of K417N mutation.^47,48^

###  Gamma (P.1)

 Gamma/P.1 lineage also known as B.1.1.28, is a more transmissible SARS-CoV-2 VOC. It is first identified in Japanese travellers returning from Amazonas State, Brazil in December 2020.^49^ Various reports showed that P.1 lineage has several S mutations including K417T, E484K, and N501Y in the RBD. Here K417T is specific when compared to B.1.351/Beta.^50,51^

###  Delta (B.1.617.2)

 This variant, which was first emerged in India in the late 2020, was spread over 163 nations by August 2021. The WHO redesignates the delta variant as a VOC from variant of interest based on its prevalence. The available evidence indicates that the Delta variant is 40%-60% more transmissible than the Alfa variant (B.1.1.7).^41^ When Delta virus infects a person’s airways, it multiplies more quickly and to a greater extent, potentially surpass the initial immune responses of the virus.^52^ Compared to Alfa variant, Delta variant processes 23 mutations with 12 mutations in S protein. T19R, L452R, T478K, D614G, P681R, and d960N, with a deletion of amino acid at position 157 and 158 are the major S gene mutations in B.1.617.2. Among these mutations the L452R and P681R S gene mutation increases the binding affinity to hACE2. The delta form with the L452R mutation may be able to avoid being attacked by CD8 T lymphocytes, which are responsible for virus eradication. P681R is another significant mutation that enhances the virus’s fusion and integration into the host cell.^53^

###  Omicron (B.1.1.529)

 This is a novel SARS-CoV-2 VOC, first reported from South Africa in November 2021 and is now being reported in various countries in the world. According to the number of mutations discovered in the S glycoprotein of SARS-CoV-2, omicron variant is the utmost divergent one.^54^ 30 mutations have been identified in the S protein of SARS-CoV-2, 15 of which are in RBD and these are accountable for the increased transmissibility, strong binding affinity, vaccine resistance and escape from immunity.^41^ According to the studies, as like the other variants, B.1.1529 also has K417N, T478K and N501Y in RBD which have an important role in the increased hACE2 binding affinity. However, B.1.1529 has 12 more mutations in RBD, but their effect has yet to be determined.^55^ In addition to these, more alterations and deletions in the genomic region and NSP 3 have also been discovered. The changes in the S protein sequence recommends that inhibition of coronavirus attachment to the host cell may not be the optimal therapy for B.1.1529.^54^

## Pulmonary manifestations of SARS-CoV-2

 The alveolar epithelium is made up of two types of cells. Type I alveolar epithelial cells (AEC1) account for 85–90% of all alveolar cells, while Type II alveolar epithelial cells (AEC2) account for the rest.^56^ They multiply and differentiate into ACE1s as progenitor cells in the alveoli, secrete a lot of surfactant protein C, which reduces surface tension and prevents alveolar collapse.^57^ They also act as a barrier for passive oxygen/carbon dioxide transport by the lungs, along with the ECs of the pulmonary microvasculature.^57,58^

 ACE2 and TMPRSS2 are highly expressed in the lungs, particularly in AEC1 cells.^59^ Hence, lung injury is the most common complication of Covid infection. Earlier research has shown that the virus suppresses the ACE2 expression in SARS-CoV as well as MERS,^60,61^ and this has now been proved in SARS-CoV 2 also.^62^ ACE2 knockout mice were used in a previous study to reveal the involvement of ACE2 in acute respiratory distress syndrome (ARDS), a pulmonary complication of COVID-19. According to the work done by Yumiko and colleagues, ACE2 knockout mice had showed a highly severe sickness in 3 separate models of ARDS: acid aspiration-induced ARDS, endotoxin-induced ARDS and peritoneal sepsis-induced ARDS, as compared to wild-type mice. ACE2 improved the clinical manifestations of lung damage in both wild as well as ACE2 knockout mice.^61,63^ This explains why ACE2 protects against lung injury. Evidently, The renin-angiotensin system is also dependent on its activity, serving it as a protective factor by generating Angiotensin 1–7 from Angiotensin II, which combats the influence of Angiotensin II by enhancing vasodilatory, anti-inflammatory, and antioxidant effects. ACE2 is recently shown to protect pressure-overload-induced heart failure in a transverse aortic constriction model of ACE2 knockout mice.^61,62^

 COVID-19 infection in the lungs progresses through four morphological stages: (a) an early stage characterised by oedema, epithelial damage, and capillarity; (b) an exudative stage characterised by diffuse alveolar damage that lasts for 1–7 days; (c) an organising stage that lasts for few weeks; and finally (d) a fibrotic stage that lasts for several weeks or even months.^64^ AEC2 cells, which overexpress ACE2, may be the primary mediators of the innate pro-inflammatory response of lower respiratory tract towards SARS-CoV-2 infection. AEC2 cells function as epithelial immune cells, and they are able to generate TNF-α, IL-6, IL-1b, MCP-1, GM-CSF, other cytokines and growth factors. Although uninfected lung cells also release pro-inflammatory cytokines at high levels, infected ACE2 and lung cells do so at higher quantities.^65^ AEC1 cells may, however, also have some effect in the innate immune response, according to some findings.^66,67^ The production of pro-inflammatory cytokines will result in a ‘cytokine storm’, as well as mucous hypersecretion,^68^ with around 10-fold increased mRNA levels of IL-6 in infected type-II cells. SARS-CoV-2 infection is more infective in the upper respiratory tract, especially in the nasal epithelium, which expresses ACE2.^58^ The greatest ACE2 levels are seen in the epithelium of nasal cavity and subsequently decline from the proximal airways to the distal lung. ACE2 and TMPRSS2 expression is observed in ciliated and secretory cells across the airway epithelium, allowing infection to spread further.^58,69^ In extensively infected AEC2 cell models, gene expression profiling indicated an apoptotic hallmark and a significant downregulation of AEC2-specific genes as well as the surfactant proteins,^70^ that may induce alveolar collapse, significant tissue damage, and scaring.^71^

## Viruses and apoptosis

 Viruses are the most infectious organisms lacking their own metabolic process and utilises host cell machinery for multiplication. Viral infections have varying degrees of severity on host cells. Apoptosis is the human cell death machinery, has a central role in viral infections. An apoptotic cell death programme can provide host defence against viral infection by inhibiting viral proliferation.^14,72^ An initial host defence response to viral infection is characterised by the destruction of virus infected cells by lymphocytes like natural killer (NK) cells and when the virus has entered the cell, it will trigger a second host defence mechanism, involving the generation of a family of cytokines called interferons. Interferons work by activating cytotoxic T lymphocytes to produce a number of intracellular genes that either specifically prevent viral replication, or aid apoptosis.^73^ Apoptotic cell death induced by viruses may aid virus clearance or cause virus-induced tissue harm or illness. Disease induction by viral infection is associated with apoptosis, either by an increased or decreased apoptosis. Some viruses are encoded with anti-apoptotic proteins that prevent apoptotic cell death, whereas others introduce viral suicide genes to the host cell and induce cell death programs, which can also be caused by an immune response.^14,72^

 Apoptosis is a cascade of energy-dependent molecular events that cells initiate in response to variety of stimuli, as well as the degradation of cellular proteins. The two key routes of apoptosis are extrinsic and intrinsic. Cell shrinkage, nuclear condensation, and plasma membrane blebbing are the main morphological characters of apoptosis.^13,74,75^ Extracellular signals activate extrinsic apoptotic pathway, also known as the death receptor pathway. This pathway cannot be initiated without the interaction of a ligand and a receptor. The two cell surface death receptors are Fas-ligand receptor and tumour necrosis factor receptor. Binding of the ligand activates the receptor and results a protein complex formation including the death receptor, adaptor protein, and procaspase 8. This activates the intracellular protease caspase 8 and cleaves effector caspase 3 and 7.^74,75^

 Intrinsic apoptosis is a type of apoptosis that is not mediated by a receptor and is also known as mitochondrial pathway of apoptosis as it is the inner mitochondrial membrane that is primarily involved in it with formation of mitochondrial permeability transition pore. Following the direct action of apoptotic stimuli on cell targets, there will be a reduction in mitochondrial membrane potential and the release of cytochrome c into the cytosol from the intermembranous space. The released cytochrome c will bind with Apaf-1 (apoptosis protease activating factor-1), triggering procaspase-9, which will then form “apoptosome” and activate the initiator caspase-9, which will then activate the executioner caspase 3 and cause apoptosis. The pro-apoptotic proteins including BAX and BAK, as well as anti-apoptotic protein BCL2, and second mitochondria-derived activator of caspases (SMAC) are also a key regulator of intrinsic apoptotic pathway. BAX or BAK induces mitochondrial outer membrane permeabilization by translocation to mitochondria in response to apoptotic stimuli and allows the release of cytochrome c to mediate cell death. SMAC acts by inhibiting the activity of apoptosis inhibitor proteins.^74-78^

###  Covid-19 and apoptosis

 SARS-CoV-2 most specifically targets the ECs, type-1 and type-2 alveolar cells and bronchial cells in the early phase causing endothelial coagulopathy, endotheliitis and apoptosis. The endotheliopathy accelerates the catastrophic phase of ARDS. Following the SARS-CoV-2 infection, the host inflammatory response becomes robust and activates the ECs, leading to endothelial dysfunction due to inflammation which can be manifested by tissue oedema and procoagulant condition.^79^ Endothelial apoptosis was observed in the post-mortem lung slices of SARS-CoV-2 infected patients with severe pre-existing illness. Apoptosis is also found in bronchial as well as lung epithelial cells of Syrian hamsters and humanised ACE2 transgenic mice, during the primary exudative stage of SARS-CoV-2 infection.^80-84^

 Accessory proteins encoded in corona virus play a crucial role in interactions of virus with the host, as well as modulation of host immunity, which in turn creates a number of mechanisms that contribute to the virus pathogenicity.^85^ The genome of SARS-CoV consist of 8 ORFs encoding new accessory proteins ORFs 3a, 3b, 6, 7a, 7b, 8a, 8b and 9b whose length varies from 39-274 amino acids.^86^ Previous research has revealed that SARS-CoV encoding ORF3a can cause cell apoptosis.^72^ In recent reports, SARS-CoV-2 ORF3a has also been shown to effectively induce apoptosis. The ORF3a of SARS-CoV is a transmembrane protein with several conserved motifs that regulate SARS-CoV ORF3 subcellular location and infection and play an important role in apoptosis induction. The tyrosine-based sorting motif (YXX;a.a.160-163), cysteine-rich motif (a.a.127-133) as well as diacidic EXD motif (a.a.171-173) are the 3 major motif in ORF3a of SARS-CoV. SARS-CoV-2 ORF3a also possess an N terminus, and showing 73% of sequence similarity to SARS-CoV ORF3a. The cysteine-rich and YXX motifs are the same, whereas the EXD motif has been replaced by SGD in SARS-CoV-2 ORF3a.^85,87,88^

**Figure 2:**
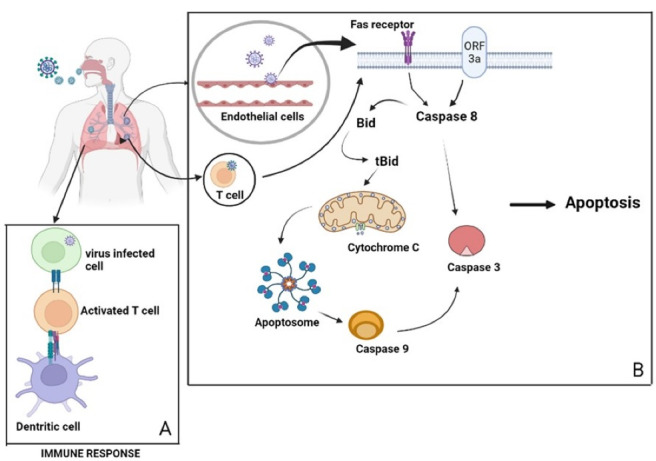
**(A)** Depicting the immune response against viral infection by the activation of T- cell. **(B)** SARS-CoV-2 enters the cell via binding to the death receptor or by the membrane fusion of ORF3a activates caspase 8 and release cytochrome c by the conversion of Bid to tBid. The released cytochome c activates the initiator caspase 9. Both caspase 8 and 9 will directly activate caspase 3 leading to the apoptosis of endothelial cells and T-cells, preventing the host immune response against viral infection

 According to a study conducted by Liu and colleagues, SARS-CoV-2 infection activates both intrinsic and extrinsic apoptosis pathways, with the EC being the primary site of apoptosis.^80^ According to Ren and colleagues research work, ORF3a membrane association is necessary for the ORF3a-mediated apoptosis promoting activity of SARS-CoV-2, meanwhile, SARS-CoV ORF3a can promote apoptosis without any membrane association. In this study, it is observed that SARS-CoV-2 ORF3a activated caspase-8 without changing Bcl-2 expression, followed by the elevated bid truncation (tBid), cleavage of caspase-9, and release of cytochrome c. Thus, these findings suggest that SARS-CoV-2 ORF3a trigger extrinsic apoptotic pathway, where the activation of caspase-8 leads to the cleavage of Bid to tBid. This triggers the mitochondrial cytochrome c release, resulting in the formation of apoptosomes and activation of caspase-9 (Figure 2).^72,85^ Caspase-8 is a key player in apoptosis, pyroptosis or necroptosis. It also stimulates pro-inflammatory cytokine production with the processing of IL-18 and pro-IL-1β, resulting in bioactive cytokine release by pyroptosis or necroptosis.^81^

 Interaction between innate and adaptive immune responses, as well as B cells, NK cells, CD4 + T cells and CD8 + T cells in the host are critical for the effective antiviral immunity against SARS-CoV-2 (COVID-19) in humans. T cells kill virus-infected cells directly and trigger the release of cytokines, which enhances the antiviral immunity of T, B, NK cells, and macrophages.^89^ Lymphopenia caused by apoptosis is a naturally occurring phenomenon in severe illnesses. Lymphopenia is directly linked to COVID-19 death, as inflammatory cytokines are thought to cause lymphocyte apoptosis.^90^ The frequency of CD4 + and CD8 + T lymphocytes is considerably reduced during COVID-19 infection and patients in severe clinical stage had much lower counts of NK cells, CD14 + monocytes, CD4 + T cells and CD8 + T cells.^89^ Studies has revealed that the CD95 and programmed cell death protein-1 (PD-1) expression in circulating CD4 + and CD8 + T cells is greater in SARS-COV-2 patients than in healthy persons. CD95 is a cell surface death receptor that is abundantly expressed in memory and effector T lymphocytes during antigen stimulation. This maintains the immunological homeostasis by triggering extrinsic apoptosis. Higher CD95 expression in CD4 + and CD8 + cells are found to be associated with decreased CD4 + and CD8 + cell numbers respectively, showing apoptosis via CD95 is a potential mechanism for COVID-19-induced lymphopenia.^91^ There is an increased rate of infection associated with the ORF3a mutation in SARS-CoV-2 and there are 18 distinct amino acid substitutions observed; five are neutral (T175I, L94F, K16N, L94I, and A72 T), whereas the rest are detrimental (Q57H, G251 V, P25L, V90F, W149L, R126 T, T176I Y109C, D155Y, T217I, D142N Y156N andK67E). This mutation generally results in the deletion of B cell epitopes and anticipated motifs identified in wild-type ORF3a.^92^ Recently, the Q57H mutation is discovered in a developing Beta variation, and the activities of these mutants are comparable to those of wildtype ORF3a in terms of inducing oxidative stress, innate immunological responses, and apoptosis and necrosis. Numerous novel ORF3a mutations have been discovered to be associated with the generation of novel viral strains.^93^

 Ren and colleagues claim that SARS-CoV-2 ORF3a has less apoptotic promoting activity compared to SARS-CoV ORF3a, implying that SARS-CoV-2 is a less pathogenic strain than SARS-CoV. ORF3a is most likely related with decreased antiviral defence mediated by apoptosis in infected cells. These traits most likely provide SARS-CoV-2 a benefit that the early infection might be moderate or even asymptomatic, increasing spreadability of the virus.^94^ According to this, there is a higher proportion of apoptotic cells in COVID19 patients with severe infection compared to those with less severe disease.^89^ It has been proposed that cytokine-mediated inflammatory cell death may be a possible mechanism explaining SARS-CoV-2-induced apoptosis.^80^ As inflammatory cytokine storm and lung injury are characteristic clinical symptoms of COVID-19 infection, viral apoptosis may provide a new hope in treating SARS-CoV-2 infection.^72^ As aforementioned, ORF3a is a unique SARS-CoV-2 accessory protein with a high expression in the infected cells and significant amount of ORF3a antibodies has been identified in affected individuals. Hence, SARS-CoV-2 ORF3a can be considered as a suitable target for the development of new therapeutic approaches for COVID-19.^95^

## Conclusion

 The existence of SARS-CoV-2 variants may be attributed to the variability of COVID-19 infections across the various geographical locations. Following a genetic analysis, it was shown that SARS-CoV-2 has changed into a less virulent strain with high incidence rate. Several accessory proteins of COVID-19 are responsible for the alveolar damage and ORF3a is one of such proteins that plays a major role in inducing apoptosis in immune cells. The association between COVID-19 and apoptosis contributes to the observed focal pathogenesis in the lungs, as well as the escalation of severity of the pulmonary complications. So, targeting SARS-CoV-2 ORF3a will be a better and effective approach to alleviate COVID-19 infection.

## Acknowledgments

 We gratefully acknowledge the Principal and HOD, Amrita School of Pharmacy, for their support and assistance.

## Competing Interests

 We wish to confirm that there are no known conflicts of interest associated with this publication.

## Ethical Approval

 Not applicable.
